# Legal, ethical, and policy challenges of artificial intelligence translation tools in healthcare

**DOI:** 10.1186/s12982-025-01277-z

**Published:** 2025-12-31

**Authors:** Hannah van Kolfschooten, Simone Goosen, Janneke van Oirschot, Barbara Schouten, Ildikó Vajda, Luna Willems

**Affiliations:** 1 University of Amsterdam, Amsterdam, The Netherlands; 2 University of Basel, Basel, Switzerland; 3 Johannes Wier Foundation for Health and Human Rights, Amsterdam, The Netherlands; 4 Patiëntenfederatie Nederland, Utrecht, The Netherlands; 5 Health Action International, Amsterdam, The Netherlands; 6 Amsterdam University Medical Centers, Amsterdam, The Netherlands

**Keywords:** Artificial intelligence in healthcare, Medical translation tools, Language barriers, Patient rights, Health data privacy

## Abstract

Artificial intelligence (AI) translation tools, such as Google Translate and ChatGPT, are increasingly used in healthcare for medical communication to overcome language barriers between patients and providers. While these tools offer accessible and efficient translation, their use raises significant legal, ethical, and policy concerns. Key patients’ rights, including the rights to privacy, informed consent, and equitable access to care, may be compromised. Current European regulations, including the EU AI Act, General Data Protection Regulation, and Medical Devices Regulation, offer only partial protection, leaving important regulatory gaps. This study employs a mixed-methods approach combining legal doctrinal analysis of EU regulatory frameworks with manual content analysis of platform terms of service. It integrates interdisciplinary perspectives from bioethics, digital health, and health communication to evaluate the implications of AI-mediated translation in clinical care. Findings reveal persistent and overlapping risks: violations of data privacy, inaccuracies in translation, bias and discrimination, and unclear liability when errors occur. To mitigate these risks, we propose targeted policy interventions, including developing guidelines for AI translation use in healthcare settings. This article contributes to digital health policy debates by identifying legal pathways to regulate AI translation tools in healthcare, ensuring their use supports patients’ rights and promotes health equity.

## Introduction

In an increasingly globalized world, European healthcare professionals face significant challenges in communicating effectively with diverse patient populations [1]. Language barriers between healthcare professionals and patients can severely impact the accessibility and quality of care, patient safety, and overall health outcomes [2, 3]. Effective communication is crucial for understanding patients’ complaints, providing accurate and timely diagnoses, ensuring patients fully comprehend their treatment options, obtaining informed consent, and shared decision making [4]. When healthcare providers and patients do not share a common language, miscommunication can have serious consequences, such as incorrect treatment, delayed care, distrust, compromised patient autonomy, and higher healthcare costs [5, 6]. However, across Europe, patients and healthcare professionals experience barriers to accessing interpreter services, for example, due to restrictive financial policies, limited entitlement to interpreters, and, in some countries and some languages, a shortage of qualified interpreters [7–9]. While there is no common right for patients in the EU to access a medical interpreter [10–12], States do need to guarantee equal access to high-quality healthcare services [13], which can be argued to include overcoming language barriers [14].

To address these challenges in mitigating language barriers, many patients and healthcare professionals are using AI tools for translation, such as Google Translate and ChatGPT [15]. These AI-powered tools are widely accessible, quick, and free to use, offering a seemingly straightforward solution to overcoming language barriers in healthcare settings. In the Netherlands, for example, research shows that 79% of migrants use Google Translate to bridge language barriers in medical settings [16]. A Canadian study shows that these tools are not only used independently by patients but are increasingly relied upon by healthcare professionals to communicate directly with patients [17]. A recent study on forty-six clinicians in the United Kingdom showed that 76.1% use online translation platforms in clinical encounters [18]. At the same time, empirical research demonstrates persistent uncertainty about the quality and reliability of digital translation tools, causing distrust and reluctance in healthcare providers to use them, especially for complex conversations [19].

In this paper, we distinguish between two types of general-purpose AI translation tools: (1) text-to-text, such as when ChatGTP is used to translate written medical instructions like discharge summaries or medication guidelines, and (2) voice-to-voice or voice-to-text, as seen with tools like Google Translate’s speech function, where spoken words are translated in real-time during medical consultations [20]. As medical-purpose translation tools do not—yet—integrate AI technologies, we excluded medical translation tools from our study.

While AI translation tools are increasingly used to overcome language barriers in healthcare, they raise significant legal and ethical concerns. These tools may impact numerous rights of patients, such as the right to access to healthcare, the right to privacy and confidentiality, the right to information, the right to autonomy, and the right to effective remedy [21]. In general, AI tools used in healthcare can introduce inaccuracies (particularly for languages of lesser diffusion), fail to capture cultural nuances, and inadvertently perpetuate biases that undermine patients’ trust in healthcare systems [22]. They also differ considerably in translation accuracy between languages. For example, a 2024 study on GPT-4’s ability to generate written discharge instructions for children showed significantly higher completeness for English than for Spanish text [15]. Moreover, when they process sensitive health information, the use of AI translation tools may pose risks to the privacy and confidentiality rights of patients, when private medical information is used to train translation algorithms [23, 24]. Another challenge concerns the right to safe and effective care: when translation errors occur, patients may suffer harm, raising questions of medical liability and the accountability of both healthcare providers and technology developers.

Most research on the use of AI for medical communication, meaning the exchange of information in verbal, written, and non-verbal form between healthcare professionals, patients, and other stakeholders in healthcare settings, focuses on AI medical chatbots rather than AI translation in medical treatment. Apart from one literature review on the societal implications of AI translation in medical settings [25], existing research primarily focuses on the technical quality of AI translation tools [26–28]. While these studies provide meaningful insights into the medical accuracy of AI translation tools, there remains a significant gap in understanding their broader legal and ethical implications for patient care. This paper aims to contribute to existing research by addressing the following question: What are the potential risks of using AI for medical translation for the rights of patients, and to what extent does the law protect against these risks?

It is important to fill this gap in light of the increasing use of AI tools in healthcare generally, and AI translation tools specifically. To do so, this paper uses a mixed-methods design. First, we conducted a manual content analysis of the internal policies of ChatGPT and Google Translate, the two most commonly used translation tools by healthcare providers. We coded them against predefined categories related to privacy, consent, liability and bias to identify patterns and divergences.^1^ Subsequently, we performed doctrinal legal analysis of relevant European regulatory frameworks, including the EU Artificial Intelligence Act, the General Data Protection Regulation and the Medical Devices Regulation, to assess how they apply to AI translation in healthcare and where regulatory gaps remain [29]. Finally, we integrated interdisciplinary insights from bioethics, health policy, and computer science to contextualise our findings and inform legal and policy recommendations. This approach ensures a comprehensive evaluation of how AI translation tools impact patient care and provides a foundation for actionable policy recommendations.

This paper is structured as follows. Section 2 explains how AI is currently deployed for translations in healthcare and its technical underpinnings. It is important to understand the technicalities to identify the legal and ethical concerns associated with AI for medical translations, as explored in Sect. 3. Subsequently, Sect. 4 analyses the applicable legal framework, focusing on the European Union (EU). Section 5 provides recommendations for policymakers, healthcare professionals, and technology developers.

## From medical interpreters to AI in medical translation

AI tools like Google Translate and other specialized translation software have become popular due to their (free) accessibility, speed, and ability to operate around the clock. AI translation tools can be broadly divided into two categories: text-to-text translation and voice-to-voice translation. Among the most commonly used tools by healthcare providers are Google Translate and ChatGPT [18, 30]. While both make use of advanced AI technologies and have opaque internal decision-making processes (“black box” systems), they deploy significantly different AI techniques. (Table 1).

Google Translate operates on Neural Machine Translation (NMT), a specialized deep learning system designed to convert text from one language to another. NMT processes entire sentences in documents rather than isolated words, with the aim of capturing the context and meaning necessary for accurate translations. It relies on parallel datasets, pairs of sentences in different languages, to learn how to translate text accurately. For voice-to-voice translation, Google Translate integrates Automatic Speech Recognition (ASR) to convert spoken language into text, which is then translated by the NMT model. The translated text is subsequently transformed into speech using Text-to-Speech (TTS) technology. This system facilitates real-time communication. Google Translate’s system is designed to learn from user feedback and adjust its models over time [31]. (Table 1).

In contrast, ChatGPT, developed by OpenAI, is built on a Large Language Model (LLM) architecture. While not specifically designed for translation, it can perform tasks requiring broader language understanding and nuanced interpretation. Unlike Google Translate, which is trained on parallel datasets that match sentences in one language with their direct equivalents in another, ChatGPT is trained on non-parallel datasets: large collections of diverse texts in many languages without one-to-one translations. This means ChatGPT does not rely on direct language pairings but instead learns general patterns of language use, allowing it to interpret meaning and context more flexibly. This allows it to handle complex or ambiguous language effectively. ChatGPT mainly focuses on text-to-text translation, where it generates output by predicting the most contextually probable sequence of words in the target language (Table 1).

**Table 1:** Key differences in ai translation tools

Feature	Google translate	ChatGPT
Primary technology	Neural Machine Translation (NMT)	Large Language Model (LLM)
Data source	Parallel corpora (aligned bilingual texts)	Non-parallel corpora (general internet text)
Specialization	Optimized specifically for translation tasks	General-purpose language understanding
Voice-to-voice support	Yes	No (text and image input only)
Context Handling	Sentence-level context optimization	Broader contextual understanding
Learning Mechanism	Real-time updates through user feedback	Periodic updates via developer retraining

Despite their growing adoption in healthcare, it is crucial to note that neither Google Translate nor ChatGPT is designed for medical purposes. ChatGPT’s Usage Policies of 29 January 2025, state that their product should not be used for “providing tailored legal, medical/health, or financial advice without review by a qualified professional and disclosure of the use of AI assistance and its potential limitations” [32]. Google Translate’s Privacy Policy of 16 September 2024 does not mention its use in medical contexts. However, both tools lack the domain-specific model training necessary to handle the complexities of medical language with consistent accuracy.

Currently, AI translation tools are being used in various ways within healthcare settings. One of the primary uses of AI translation tools is in facilitating patient consultations [33]. Voice-to-voice translation tools, such as Google Translate’s speech functionality, are frequently used during medical consultations to enable real-time communication with patients who do not speak the provider’s language. This is particularly valuable in emergency care settings, where immediate understanding is critical to making informed decisions [34]. In lower-urgency scenarios, these tools play an important role in physical as well as telemedicine consultations, helping to communicate patient histories, symptoms, and concerns effectively [35].

AI translation tools are also extensively employed for translating medical documents, such as discharge summaries, medication instructions, consent forms, and patient education materials [36, 37]. Text-to-text translation tools like Google Translate and ChatGPT offer quick translations, allowing healthcare providers to convey essential information to patients or caregivers in a timely manner, for example, post-operative discharge instructions [38]. These tools are further utilized to translate patient records and diagnostic reports for cross-border medical consultations or second opinions. Another specific example is the development of multilingual AI apps to facilitate the convening of background information in the context of rapid diagnostic testing, specifically aimed at migrant patient groups [39]. In addition to clinical applications, AI translation tools are used for administrative and operational support. Healthcare institutions rely on these tools to translate appointment reminders, administrative forms, and internal communications with non-native-speaking staff [40].

While human interpreters are not free from shortcomings, such as availability constraints, potential human error, or subjective interpretation, their work is embedded in professional, ethical, and legal frameworks that support quality control and accountability. In the Netherlands, several health laws require providers to deliver good quality care and inform patients in a language they understand. Case law confirms that this may require engaging someone fluent in the patient’s language, preferably a professional interpreter. Similar safeguards exist elsewhere, such as the NHS Interpreting and Translation Framework in the UK [10]. AI translation tools, by contrast, offer speed and convenience but lack embedded cultural mediation, real-time contextual clarification, and formal accountability structures. Thus, while both methods can introduce errors, the nature, detectability, and redress mechanisms for those errors differ significantly.

## Ethical and legal concerns in the use of AI translation tools in healthcare

The integration of AI translation tools in healthcare settings has raised critical questions surrounding the ethical and legal implications of using automated systems to communicate with patients [25]. These challenges span from concerns about privacy and data protection to questions about the lack of accuracy of translations, patient autonomy, the risk of bias in translations, and legal uncertainties surrounding liability for errors. To ground this analysis, we first summarise the results of our manual content analysis of ChatGPT and Google Translate policies across predefined categories in Table 2. These findings inform the discussion that follows, which elaborates on the potential risks and implications for healthcare professionals, healthcare institutions, and patients alike.

**Table 2:** Summary of content analysis of Google Translate and ChatGPT

Category	Google translate	ChatGPT
Data protection & privacy	States that user data may be stored and reused to improve services; limited options to opt out of data retention	States that user inputs may be reviewed and used to improve model performance unless chat history is disabled
Transparency	Provides general privacy policy with limited detail on how translation data are processed or stored; no healthcare-specific disclosures	Offers general explanations about data use and retention; does not clarify how medical information is handled or whether human review may occur
Accountability	No designated contact point or redress mechanism for translation-related errors; responsibility for safe use rests with the user	Includes general feedback and appeal options but explicitly disclaims liability for outcomes; healthcare use discouraged in usage policy
Health-specific safeguards	No mention of medical contexts or health data protection requirements	Explicitly advises against using ChatGPT for medical advice or diagnosis but provides no technical safeguards for health-related inputs

### Privacy and data protection

The use of AI translation tools in healthcare settings raises critical privacy and data protection concerns, particularly regarding the handling of sensitive patient information. AI translation tools process user inputs—often containing highly sensitive details such as medical conditions, treatment plans, or medication instructions, and identifying personal information—by transmitting them to external servers for analysis. This reliance on third-party platforms creates several privacy risks.

One significant risk involves unauthorized access and data sharing. Many AI translation tools, including Google Translate, operate on cloud-based platforms where user data may be stored and analyzed to improve their services. For example, their privacy policy states: “We use your interactions with AI models and technologies (…) to develop, train, fine-tune, and improve these models to better handle your requests, and update their classifiers and filters including for safety, language understanding, and factuality”, and: “We analyze usage of Google Translate to improve translation quality and increase the availability of Translate in more languages” [41].

According to Google’s terms of service, input data may be retained for quality enhancement purposes, which could include unintended access or sharing with third parties. It is for example stated that: “We use publicly available information online or from other public sources to help train new machine learning models and build foundational technologies that power various Google products such as Google Translate, Gemini Apps, and Cloud AI capabilities.” Furthermore, cross-border data transfers present additional challenges, as data may be processed in jurisdictions outside the European Union with different data protection standards. These transfers raise concerns about the adequacy of safeguards in place, potentially exposing patient data to weaker legal regimes. For Google Translate, there is no full opt-out from data processing when using the free web or app versions; input data may be stored and used for service improvement. For ChatGPT (free and Plus versions), inputs may be used for model training unless users adjust settings to disable chat history, but this does not guarantee zero retention.

Another pressing issue is the lack of user awareness and consent. Patients and healthcare professionals are often unaware that using AI translation tools for medical communication involves sharing sensitive information with external service providers. This lack of awareness undermines one of the main aims of data protection legislation: enhancing individual control over (sensitive) personal data. Moreover, the absence of explicit mechanisms to inform users about how their data will be processed risks breaching privacy regulations and eroding patient trust.

The privacy policies of AI translation tools reveal additional gaps in compliance with healthcare data protection standards. Google Translate’s policy, for example, states that inputs may be stored for service improvement, with limited options to opt-out. Similarly, OpenAI’s ChatGPT explicitly disclaims responsibility for misuse, acknowledging that inputs may be temporarily stored for quality assurance but without offering healthcare-specific safeguards. These limitations highlight the challenges of relying on general-purpose tools for medical communication.

Real-world incidents underscore the severity of these risks. For instance, in 2017, a Norwegian energy company experienced a significant data breach when sensitive information translated through an online AI translation tool was inadvertently exposed online [42]. In 2023, nine million patients in the US had their health data stolen in a cyberattack on a company that converted audio recordings of medical consultations to text [43]. A 2019 study assessing the use of Google Translate for emergency department discharge instructions, found that 8% of Chinese sentence translations had the potential for significant harm [44].

### Patient autonomy, the right to information, and informed consent

Patient autonomy is a foundational principle in healthcare, ensuring that individuals have the right to make informed decisions regarding their health. For informed consent to be valid, patients must fully understand their diagnoses, treatment options, and associated risks. However, the use of automated translation tools like Google Translate can compromise this process, especially in medical contexts where terminology and explanations are complex.

For example, migrants often rely on Google Translate to translate written medical instructions, prescriptions, and doctors’ letters due to its accessibility and ease of use. However, the potential for inaccurate translations raises serious ethical concerns. Mistranslations may lead to patients misunderstanding their medical conditions or treatment options, resulting in compromised informed consent. This not only undermines patient autonomy but, if healthcare providers use Google Translate to translate medical documentation, they are also placed in ethically precarious situations where they may inadvertently mislead patients.

Legally, the issue of informed consent is further complicated by language barriers, as highlighted in several rulings by the European Court of Human Rights (ECHR). In the case of *V.C. v. Slovakia*, a Romani woman was sterilized without her full and informed consent because the medical information was not adequately communicated in a language she could understand. The ECHR ruled that this violated her rights under Articles 3 and 8 of the European Convention on Human Rights. Similarly, in *Mayboroda v. Ukraine*, a patient underwent kidney removal without clear informed consent due to inadequate communication. These cases emphasize the necessity for healthcare providers to ensure that all patients receive medical information in a language they fully understand, underscoring the risks posed by using AI translation tools.

### Bias and discrimination

The accuracy of AI translation tools is closely linked to bias and discrimination. Differences in accuracy between languages, dialects, and accents are often the result of imbalances in training data, meaning that certain groups systematically receive lower-quality translations. This can directly affect patient safety, autonomy, and trust, and may amount to indirect discrimination.

AI translation tools are trained on vast datasets that may reflect societal and cultural biases, which can permeate their outputs. Language reflects the complexities of cultural, socioeconomic, and historical contexts, and automated translation systems—trained on pre-existing datasets—may inadvertently replicate or magnify these biases [45]. AI translation tools are trained on vast datasets that may reflect societal biases, which can permeate their outputs. For example, translations might unintentionally perpetuate stereotypes or exclude culturally specific medical terminology, health conditions, or practices. Such biases can inadvertently influence patient-provider interactions, especially in healthcare settings where trust is essential [46]. Moreover, the dependence on machine translation brings up significant ethical questions about whose perspectives are prioritized and whose are disregarded in the pursuit of greater linguistic accessibility [47].

Moreover, some languages are more prone to gender biases in AI translations, particularly when translating from languages that do not explicitly indicate gender in their grammar, such as Finnish, Hungarian, and Turkish [48]. AI systems often default to male pronouns and stereotypically male attributes when translating into English, while female pronouns are more likely to be associated with adjectives that emphasize nurturing traits. For example, research shows that Google Translate tends to favor male pronouns and uses stereotypically gendered language, with adjectives like “strong” and “brave” associated with men and “shy” and “happy” with women. For example, a female patient describing cardiovascular symptoms in Turkish could have her account translated in a way that emphasizes emotional or psychological causes, while a male patient’s account might emphasize physical severity, potentially influencing diagnostic and treatment decisions.

Additionally, translation accuracy varies significantly across languages. AI translation tools tend to perform less effectively with non-European languages, such as Farsi and Armenian, which can have error rates as high as 32–45% [30, 49]. Inaccuracies of this scale in critical medical communication can have serious consequences, for instance, mistranslating “take twice daily” as “take two at once” could lead to an overdose, and an inaccurate allergy warning could result in exposure to a life-threatening allergen. Furthermore, people speaking in a dialect or heavy accent often get less accurate results in the speech translation tools [50].

From a legal perspective, the use of biased AI translation tools can expose healthcare institutions to allegations of discrimination, potentially violating anti-discrimination laws. When patients receive substandard care due to inaccurate or biased translations, healthcare providers may face legal repercussions, especially if these disparities contribute to worse health outcomes for certain patient groups. This issue is particularly concerning for non-native speakers and marginalized communities who are already at a disadvantage in accessing healthcare. If AI tools consistently deliver lower-quality translations for these groups, it not only risks medical errors but also perpetuates health inequities by systematically disadvantaging those who rely on these technologies the most. As a result, the use of biased AI translators can widen existing disparities, undermining efforts to achieve equitable healthcare and violating the fundamental right to equal treatment.

### Challenges with medical liability for AI translation errors

While the previous sections discuss specific risks posed by AI translation tools, this section addresses the broader challenge of accountability when those risks materialize. Liability is essential to ensuring that violations of privacy, consent, or equality are actionable. However, if a mistranslation results in patient harm, such as incorrect medication instructions or misunderstandings about surgical procedures, determining who is responsible becomes highly ambiguous. In many cases, liability may be shared between different parties. For instance, the hospital or healthcare provider who used the AI tool could be held accountable for failing to ensure the accuracy of the information conveyed. At the same time, there could also be grounds for holding the AI system developer liable, especially if the error was due to flaws in the algorithm or biases in the training data used to develop the tool. Beyond individual patient harm, AI translation errors in healthcare could result in mass damage in scenarios such as miscommunication during public health emergencies, such as isolation guidelines, causing confusion, non-compliance, and unequal protection for non-native-speaking communities.

Currently, the legal frameworks do not clearly delineate the boundaries of liability in cases where AI systems are involved. If a healthcare provider relies on a tool like Google Translate for medical communication and an error leads to adverse outcomes, it is often unclear whether the fault lies with the healthcare professional, the healthcare institution, or the AI technology provider. This legal ambiguity can result in costly disputes and create uncertainty for healthcare organizations that utilize AI translation tools, potentially deterring their adoption in situations where they might be beneficial [51].

Legal precedents set by the European Court of Human Rights, such as in *V.C. v. Slovakia* and *Mayboroda v. Ukraine* (see Sect. 3.2), highlight the significant legal risks when patients are not adequately informed due to language barriers. If healthcare providers rely on AI translation tools like Google Translate for obtaining informed consent and these tools produce mistranslations, they could face similar legal challenges. In such cases, healthcare providers may be held accountable if patients suffer harm due to a lack of clear communication in a language they understand, potentially violating their rights under the European Convention on Human Rights.

To mitigate these risks, healthcare providers should take a cautious approach, using AI translation tools primarily for non-critical communication [27]. In high-stakes situations, such as conveying surgical instructions or discussing complex treatment options, it is advisable to rely on human interpreters or specialized medical translation tools that are specifically designed for healthcare use. Additionally, healthcare organizations should establish clear internal guidelines and provide staff training on the limitations of AI tools. They should also be transparent towards patients on whether AI tools were used for medical translation. By doing so, they can reduce potential legal liabilities and better protect patient safety while leveraging the benefits of AI translation technology in appropriate contexts.

## Legal regulation of AI translators in healthcare

As AI-powered translation tools, like Google Translate and ChatGPT, become more commonly used in healthcare settings to overcome language barriers, it is essential to examine their legal regulation. This section explores how the EU’s existing regulatory frameworks—including the Medical Devices Regulation (MDR), the EU Artificial Intelligence Act (EU AI Act), and the General Data Protection Regulation (GDPR)—apply to these technologies. Additionally, we will address the challenges surrounding liability if AI translators produce errors in medical contexts.

### EU medical devices regulation: limited applicability to AI translators

The MDR, which came into full effect in 2021, sets strict standards for the safety and efficacy of medical devices used within the EU. Under the MDR, a device must meet specific regulatory requirements if it is intended for medical purposes, such as diagnosis, prevention, monitoring, or treatment of diseases.

However, AI translation tools generally do not fall under the scope of the MDR. The MDR applies only to products explicitly marketed as having a medical intention. Since Google Translate, ChatGPT, and similar tools are general-purpose technologies not specifically designed for healthcare use, they are not classified as medical devices under the MDR. This lack of classification means they are not required to undergo the rigorous testing and certification processes that medical devices must comply with [52].

Despite their increasing use in healthcare settings, particularly by doctors and nurses to communicate with non-native speakers, the absence of a medical-specific intention excludes tools like Google Translate and ChatGPT from the MDR’s scope. Therefore, these tools are unregulated from a medical safety perspective, which can be problematic given the potential for translation errors in critical healthcare situations.

### EU AI Act: Limited rules for general AI translators

The EU AI Act, set to become fully enforced in 2025, introduces a risk-based framework for regulating AI systems. The Act classifies AI systems into four categories: minimal risk, limited risk, high risk, and unacceptable risk, with varying levels of regulatory obligations.

AI translation tools are generally classified as “Limited Risk” under the EU AI Act. This categorization is based on the fact that these tools are designed for general-purpose use and not for use in healthcare. Moreover, they are considered to not pose significant direct risks to health or safety. As a limited-risk AI system, AI translation tools are subject to basic transparency obligations, such as informing users that they are interacting with an AI system. However, it is not subject to the more stringent requirements reserved for high-risk AI systems, such as those used in medical diagnostics or autonomous vehicles [53].

The EU AI Act does recognize the potential for general-purpose AI systems, like translation tools, to be used in high-stakes contexts, such as healthcare. However, it primarily relies on transparency measures and voluntary codes of practice to address risks, rather than imposing strict regulatory controls. This limited regulatory oversight raises concerns, particularly when these tools are used in critical healthcare situations where errors could lead to adverse outcomes [54].

### GDPR: Data privacy and security for AI translators

The GDPR, which governs data protection and privacy within the EU, directly applies to AI translation tools when they are used to process personal data. Since healthcare data is classified as “special category data” under the GDPR, its handling requires heightened safeguards to protect patient privacy. However, when healthcare providers use tools like Google Translate or ChatGPT to translate patient information, significant data privacy concerns arise.

A key issue concerns the legal basis for processing. While the initial processing of patient data is generally lawful as necessary for medical treatment, secondary uses, such as translation through external AI platforms, may fall outside that scope.^2^ Failure by the healthcare provider to inform the patient can undermine patient trust and violate GDPR requirements [55].

Additionally, the way AI translation tools handle data storage and processing raises concerns. For example, in the case of Google Translate, Google’s terms of service mention that data entered into Google Translate may be used to improve the platform’s services, which could include storing user inputs. Data retention poses risks as healthcare providers may inadvertently expose sensitive patient information to external entities. The GDPR mandates that organizations using such tools implement robust safeguards to prevent unauthorized access and misuse of personal data, ensuring that any processing is secure and compliant with privacy laws [56, 57].

Healthcare providers and healthcare organizations bear the responsibility of ensuring that any AI translation tools they use comply with GDPR standards. This includes conducting Data Protection Impact Assessments (DPIAs) to identify and mitigate potential risks associated with the use of AI translation systems in healthcare. By proactively assessing these risks, healthcare providers can better protect patient confidentiality and maintain compliance with regulatory requirements, thereby avoiding legal repercussions and safeguarding patient trust.

Some hospitals and healthcare providers explicitly state on their websites that Google Translate is used in the course of medical consultations, or they provide extra information for patients on the purposes for which AI translation tools are used in the organization (e.g., for translating the provider’s website or for sending medical letters).^3^

## The way forward: policy recommendations

To address the ethical, legal, and policy concerns associated with the use of AI translation tools in healthcare, a multi-faceted approach is essential. The following recommendations focus on establishing guidelines, investing in specialized tools, and enhancing data protection measures to ensure that AI technologies are used responsibly and effectively in healthcare settings.

### Establishing guidelines for translation tools

Healthcare institutions must establish clear, evidence-based, and compliant guidelines to govern the use of translation tools. These guidelines should focus on critical areas such as privacy, accuracy, and informed consent to protect patient rights and ensure safe and effective communication in clinical settings. The first step is to develop general guidelines for mitigation of language barriers in healthcare. This includes considering whether patients have sufficient health literacy to understand complex medical information. The complexity of care, such as whether a conversation involves diagnosis, treatment options, or risks, should inform the choice of translation support, whether AI-based or human.

Crucially, guidelines must go beyond instructing how to use AI tools correctly: they must help clinicians determine whether AI translation is appropriate in the first place, or whether the current gold standard, a professional medical interpreter, should be used. While some healthcare interactions may initially appear straightforward and non-sensitive, and could therefore be suitable for AI, sensitivity can emerge unexpectedly, and the threshold between sensitive and non-sensitive content is often ambiguous. Given these uncertainties, decisions about when to use AI versus human translators should be guided by clear protocols. Clinical protocols should be aligned with existing national standards and legal obligations regarding interpreting services, such as those in the Netherlands and the United Kingdom [10], which recognize professional interpreters as essential in high-stakes or complex interactions. Another essential aspect of these guidelines is the active involvement of patients in deciding what kind of language support will be used. Patient-centered care requires that healthcare professionals discuss and agree with patients on the most suitable form of communication support, based on the patient’s needs, preferences, and the context of care [58].

Comprehensive training of healthcare providers is also key. Under Article 4 of the EU AI Act, providers and deployers of AI systems must ensure that their staff achieve an adequate level of AI literacy, particularly when using high-risk systems [53]. This training should include recognizing situations where a translation tool may be inadequate and knowing when to involve professional human translators. Emphasizing the need to verify translations, especially in high-stakes situations such as surgical consents or medication instructions, is vital since errors in these contexts can have severe consequences. For example, in the Netherlands, a group of researchers, healthcare providers, patients, translators, and other stakeholders, is developing a guideline to assist professionals, patients, and clients in choosing an appropriate method when faced with language barriers [58].

Guidelines should also include robust verification processes to ensure the accuracy of AI-generated translations. For instance, employing a “teach-back” method, where patients are asked to repeat their understanding of medical instructions, can help confirm that critical information has been accurately conveyed and fully comprehended [59]. This approach not only improves communication but also enhances patient safety by reducing the likelihood of misunderstandings.

### Investing in specialized medical translation tools

General-purpose AI translation tools like Google Translate are not specifically optimized for healthcare settings and often lack the necessary safeguards for handling sensitive patient data. This presents significant risks, especially when these tools are used in contexts that require high levels of accuracy and confidentiality. Therefore, there is a pressing need for organizations in the healthcare sector to invest in specialized medical translation tools that are designed to meet the unique demands of healthcare communication.

Developing and deploying domain-specific tools that are tailored to medical contexts, can provide greater accuracy and enhanced privacy protections compared to general-purpose AI tools [20]. If specialized platforms are trained on healthcare-specific terminology, this makes them better equipped to handle the complexities of medical language, thereby reducing the risk of mistranslations that could compromise patient safety. Additionally, many general-purpose AI tools operate on cloud-based platforms, which may store user data and thus pose privacy risks. A solution would be to invest in offline or locally hosted translation tools, including open-source systems and institutionally managed models that operate within secure hospital networks. These tools could be integrated into clinical information systems, allowing translation to occur without transmitting data to external servers. By investing in offline translation solutions and privacy-by-design solutions, healthcare organizations can maintain control over patient data and ensure compliance with stringent data protection regulations like the GDPR. Offline tools not only safeguard patient confidentiality but also function effectively in areas with limited internet access, making them particularly valuable in remote or resource-constrained environments.

Furthermore, there is a significant opportunity for governments, healthcare organizations, and insurance companies to collaborate with technology companies in the development of AI translation tools that are specifically tailored to healthcare needs. These public–private partnerships can drive innovation while integrating ethical and legal standards into the design and deployment of new technologies. Importantly, ownership and long-term control over these tools should rest with healthcare organizations. Over-reliance on commercial technology providers creates structural vulnerabilities: tools may be withdrawn, rebranded, made subscription-based, or updated in ways that do not align with clinical priorities. This can disrupt continuity of care and undermine trust in digital infrastructure. Moreover, commercial incentives may deprioritize languages or user groups that are less profitable, exacerbating health inequities [60]. To ensure continuity of service and alignment with healthcare values, healthcare institutions must lead the development and governance of AI translation solutions. Interdisciplinary collaboration between technologists, ethicists, and healthcare providers is also crucial for creating AI translation tools that prioritize patient safety. By fostering these partnerships, the healthcare sector can leverage technological advancements while ensuring that AI tools are used responsibly and ethically.

### Enhancing data protection measures

The use of AI translation tools in healthcare must prioritize the protection of patient data, given the sensitive nature of medical information. Ensuring compliance with data protection regulations is essential to prevent breaches that could expose patient information to unauthorized entities. Healthcare organizations should implement robust data security protocols to ensure that these AI translation tools do not store or share sensitive patient data. This involves using tools that are fully compliant with regulations like the GDPR, which mandates that healthcare providers take all necessary precautions to safeguard patient information [61]. For instance, as part of a broader compliance strategy, healthcare providers could publicly acknowledge on their websites the use of AI translation tools and outline their data policies. Such transparency must be accompanied by stronger measures, such as offering alternatives to patients who do not consent to AI use, ensuring secure data processing, and maintaining compliance with applicable regulations.

Furthermore, transparency in data handling practices and quality control is crucial. Providers of AI translation tools must clearly communicate how they collect, store, and utilize data. Healthcare organizations should only adopt tools that have explicit and transparent terms of service, ensuring that patient data is not repurposed for secondary uses without the patient’s explicit consent. To maintain high standards of data protection, healthcare institutions should conduct regular audits of the AI tools they use [62]. These audits help ensure ongoing compliance with privacy regulations and can identify potential vulnerabilities in data handling practices. Continuous monitoring and periodic updates to data security measures are essential as AI technologies continue to evolve, helping healthcare providers stay ahead of emerging threats and protect patient confidentiality effectively [63].

We recognize that implementing these recommendations requires financial and logistical resources that may be limited in many healthcare settings. A phased approach may therefore be more realistic, starting with high-priority situations (e.g., emergency care, communication required for obtaining informed consent before surgery) and gradually expanding to broader use cases.

## Concluding remarks

The integration of AI translation tools in healthcare brings forth both opportunities and challenges. These technologies have the potential to improve accessibility, increase efficiency, and mitigate language barriers within clinical settings. However, their deployment also raises significant ethical and legal concerns, particularly in relation to discrimination, privacy, and informed consent. To fully realize the benefits of AI while minimizing associated risks, healthcare organizations must proceed with caution. This requires the establishment of robust guidelines, investment in specialized medical translation tools, and the implementation of stringent data protection measures to safeguard patient rights. The objective should not be to entirely replace human translators, but rather to utilize AI as a complementary tool that enhances, rather than compromises, the patient experience. By prioritizing responsible innovation, healthcare providers can ensure that AI translation tools are employed in a manner that promotes equitable and effective care for all patients, regardless of linguistic differences.

## Data Availability

No datasets were generated or analysed during the current study.

## Footnotes

1 NB: while there are specific medical translation tools on the market, healthcare professionals seem to prefer the use of these freely available tools.

2 See Article 9 GDPR.

3 See for example Catharina Ziekenhuis in the Netherlands: https://www.catharinaziekenhuis.nl/vertaalmogelijkheid-voor-catharina-website/.
